# Rethinking assessment for practice readiness: policy implications of the OSCE–practice mismatch in Malaysia

**DOI:** 10.1080/20523211.2026.2735132

**Published:** 2026-09-30

**Authors:** Athirah Bakhtiar, Angelina Lim, Li Ling Yeap, Daniel Malone, Han Yin Lim, Sunanthiny Krishnan

**Affiliations:** a School of Pharmacy, Monash University Malaysia, Selangor, Malaysia; b Faculty of Pharmacy and Pharmaceutical Sciences, Monash University, Parkville, VIC, Australia; c Department of Cardiovascular Sciences, University of Leicester, Glenfield Hospital, Leicester, UK

**Keywords:** Objective Structured Clinical Examination (OSCE), mystery shopping, workplace-based assessment, pharmacy education, health workforce policy, Malaysia

## Abstract

**Background:**

Experiential learning is central to pharmacy education, yet the translation of clinical skills from simulated examinations to actual practice remains under-researched. This study utilised mystery shopping to evaluate how well clinical competencies transfer when students move from university Objective Structured Clinical Examinations (OSCEs) to independent community pharmacy placements, shedding light on systemic barriers to clinical care integration.

**Methods:**

Using a sequential explanatory mixed-methods design, 57 third-year undergraduate pharmacy students were covertly evaluated during their placements by mystery shoppers reenacting clinical scenarios identical to those used in the primary-care component of their university OSCEs. Scores were compared using Wilcoxon signed-rank tests and Spearman’s correlation. Additionally, semi-structured interviews explored students’ perspectives on the performance gap and the systemic obstacles that prevent successful skill transfer.

**Results:**

Real-world performance was markedly lower than examination scores, with mystery shopping scores dropping to a median of 0% versus 91.67% in the OSCE (*p* < 0.001). No significant correlation existed between simulated success and independent execution (r_s_ = 0.14, *p* = 0.2934). Qualitative analysis identified three main drivers for this mismatch: the disconnect between structured examinations and fluid retail practice; environmental distractions; and restrictive public expectations that actively discouraged thorough clinical counselling.

**Conclusion:**

Simulated clinical competence does not reliably translate to independent practice. This performance drop illustrates how national policy and retail realities constrain a student's actual clinical output. Integrating independent workplace assessments, such as mystery shopping, may provide a more direct, unsupervised measure of readiness. Bridging this gap will likely require coordinated efforts among academic institutions, practising pharmacists, and regulatory bodies. These findings may offer preliminary insights relevant to other developing health systems navigating similar transitions in clinical pharmacy practice, pending further research across diverse settings.

## 1. Introduction

The modern landscape of pharmacy practice demands that educational models look far beyond classroom theory to prioritise the development of adaptable clinical skills (Gazzaz & Wali, 2026; O’Sullivan & Sy, 2017). As the pharmacist's role grows increasingly sophisticated, encompassing an expanding scope of practice globally (International Pharmaceutical Federation [FIP], 2025), including value-added services such as medication therapy management and health screening within the Malaysian context (Chu et al., 2024), graduating cohorts must be practice-ready, capable of autonomous, adaptive decision-making in unpredictable healthcare environments (Gazzaz & Wali, 2026; O’Sullivan & Sy, 2017). Achieving this degree of readiness is no longer just an educational ideal; it is a critical health policy priority given the patient-safety risks that emerge when training environments fail to mirror actual practice conditions (FIP, 2025).

This transition is uniquely complex in Malaysia, where the healthcare system is actively pivoting from a traditional, dispensing-focused retail model to patient-centred clinical care. Historically, community-based pharmaceutical services in the country have lagged behind international clinical standards (Loh et al., 2021), and local pharmacists frequently report reduced clinical decision-making autonomy compared to their Western counterparts (Nik et al., 2016). This systemic constraint is particularly striking because Malaysian pharmacy programmes provide rigorous, structured training in problem-solving and triage (Lee et al., 2025). To help bridge this classroom-to-clinic divide, curricula have substantially integrated experiential learning (O’Sullivan & Sy, 2017). Grounded in Kolb’s Experiential Learning Theory, these hands-on placements immerse students in real-world pharmacy environments, challenging them to transform academic knowledge into authentic clinical practice (Kolb, 2014). While Kolb's Experiential Learning Theory explains why direct practice experience is essential for developing genuine clinical competence, it does not address whether prior assessment performance can be trusted to predict that competence in unsupervised, real-world settings, a question Kane's validity framework is designed to answer (Kane, 2013).

Yet, despite the widespread use of high-fidelity simulations, such as OSCEs designed to closely replicate real clinical encounters, to build initial competence, an important gap remains in the literature: there is very little empirical data on how these skills translate when a student transitions to independent practice. Unpacking this transfer of learning is crucial in a developing practice landscape like Malaysia’s, where building professional autonomy can strengthen student confidence, preceptor trust, and public reliance on pharmacy services. Most existing research measures success through student self-assessments or controlled laboratory performance. Consequently, there is an urgent need for robust evaluation tools that measure clinical acumen under the true cognitive load of an unscripted pharmacy environment. Mystery shopping, a method where a trained individual covertly interacts with a specific subject of interest (in this case, the student) to objectively evaluate practice quality, offers an innovative solution (Collins et al., 2017; Harden et al., 1975). By acting as a non-disruptive bridge to traditional clinical rotations, it provides a transparent lens on clinical reasoning and communication without compromising patient safety.

Adopting naturalistic assessments remains logistically challenging due to constrained placement capacities and valid concerns over student performance in high-stakes live settings. To manage these pressures, institutions depend on Objective Structured Clinical Examinations (OSCEs) for their exceptional reliability, standardisation, and logistical control (Kardong-Edgren et al., 2023). However, evidence suggests that performance demonstrated in simulated environments may not fully correspond with performance in the workplace (Prudhomme et al., 2020), raising questions about the extrapolative validity of OSCEs. As framed by Kane’s validity framework (Kane, 2013), assuming that high performance in a structured examination automatically predicts competent execution in an unscripted live encounter represents a largely untested leap at the extrapolation inference level.

To address this extrapolation gap, workplace-based assessments (WBAs) like mystery shopping have emerged as effective strategies for evaluating practitioners in situ (Collins et al., 2017). Because the evaluation is completely unobtrusive, it minimises the ‘Hawthorne effect’, the observer bias that artificially inflates student performance during standard clinical examinations (Paradis & Sutkin, 2017). Furthermore, when these covert encounters are intentionally mapped to identical case scenarios from previous university OSCEs, they create a unique opportunity to track the direct transfer of learning under identical clinical parameters.

While a recent Australian study utilising this design revealed that standard OSCEs significantly overestimate a graduate’s real-world readiness (Lim et al., 2024), the formal integration of mystery shopping within Asian pharmacy education and workforce policy remains virtually undocumented. Regional health system structures, distinct cultural dynamics, and regulatory variations mean that the feasibility and interpretation of covert audits require localised investigation (Chu et al., 2024; Miller & Goodman , 2016). Accounting for these unique systemic differences, this study examines how clinical competence transfers from controlled simulation to independent community practice in Malaysia, with the aim of shedding light on the systemic barriers to clinical care integration faced by graduating students transitioning into practice.

## 2. Methodology

### 2.1. Participants and setting

The OSCE serves as a pivotal assessment within the four-year Bachelor of Pharmacy (Honours) programme at Monash University Malaysia. Operating as a vertically integrated curriculum, this programme was developed in parallel with, and modelled on, the vertically integrated curriculum design implemented at the Australian parent campus (Malone et al., 2021), with the intention of aligning curriculum structure and learning outcomes across both campuses. The university OSCE assessment specifically, including content, format, and delivery, is standardised across both the Malaysian and Australian campuses, consistent with the assessment design used in the referenced Australian mystery shopping study (Lim et al., 2024), which shares co-authorship (DM) with the present study. OSCEs are embedded across all year levels as both formative and summative assessments to support progressive competency development.

For this study, third-year pharmacy students underwent a summative OSCE at the end of Semester 1, concluding an intensive ten-week module on infectious diseases (Lim et al., 2023). The module curriculum addresses critical clinical spaces, such as antimicrobial stewardship (AMS), respiratory and skin infections, and the management of chronic viral hepatitis and HIV. This curriculum content, including prescription validity screening and identification of drug-related problems, is taught extensively prior to placement; however, the two-week community pharmacy rotation represents students’ first opportunity to apply this knowledge independently during a covert, unsupervised assessment, in a live clinical encounter. Prior to this rotation, students had also completed a shorter community pharmacy placement earlier in the programme, under normal preceptor supervision throughout, providing some prior exposure to the retail practice environment, though not to independent, unsupervised practice specifically. The final examination format comprised three distinct assessment stations: a hospital-based clinical review, a primary care environment, and a closed-book product counselling assessment.

The primary care station was designed to replicate the clinical demands of a community pharmacy setting. Within this simulation, students were required to manage over-the-counter (OTC) consultations, interpret prescription requests, or conduct acute symptomatic triage. Student performance was evaluated using an analytical checklist designed to measure objective clinical accuracy and procedural thoroughness (see Supplemental Appendix 1). To preserve the operational security of the university’s institutional assessment bank and ensure the validity of upcoming examination cycles, the precise case scripts cannot be published in full; however, a comprehensive summary of the case scripts and their mapped clinical competencies is provided in Supplemental Appendix 2.

Subsequently, during Semester 2 and within 12 weeks of the initial examination, the identical student cohort progressed to a mandatory two-week experiential rotation across accredited community pharmacies. Throughout this practice block, students were supervised directly by registered pharmacist preceptors. Real-world clinical performance in practice was documented using Entrustable Professional Activities (EPA) metrics recorded inside longitudinal student logbooks. These EPA scores served as a direct measure of supervisor-assessed workplace performance, focusing specifically on primary-care triage and frontline clinical interventions (Haines et al., 2017). This structured data provided a clear longitudinal benchmark to contrast against the students’ prior simulated examination results. The specific EPA framework, complete with level descriptions and evaluated practice domains, is detailed in Supplemental Appendix 3.

### 2.2. Study design and participant recruitment

This study adopted a two-phase, sequential explanatory mixed-methods approach. The initial quantitative phase examined performance across three assessment modalities, while the subsequent qualitative phase used semi-structured interviews to explore student reflections and provide context for the quantitative findings.

All third-year pharmacy students spanning the 2022 and 2023 academic cohorts (n = 237) who had successfully completed the Semester 1 infectious disease unit and its accompanying OSCE were invited to enrol. Community pharmacy placements, and therefore participant availability, occur once per academic year at year-end; data collection accordingly commenced with the 2022 cohort's placement and continued through the 2023 cohort's placement under an identical protocol, checklist, and case content, to achieve an adequate sample size for robust statistical comparison. Out of this eligible pool, 159 students (67%) provided formal written consent (see Figure 1). This participation rate may partly reflect concerns about negative evaluation (Downing et al., 2020), where certain candidates may have perceived a covert assessment exercise as a risk to their professional reputation despite rigorous institutional guarantees of data confidentiality.

**Figure 1.**
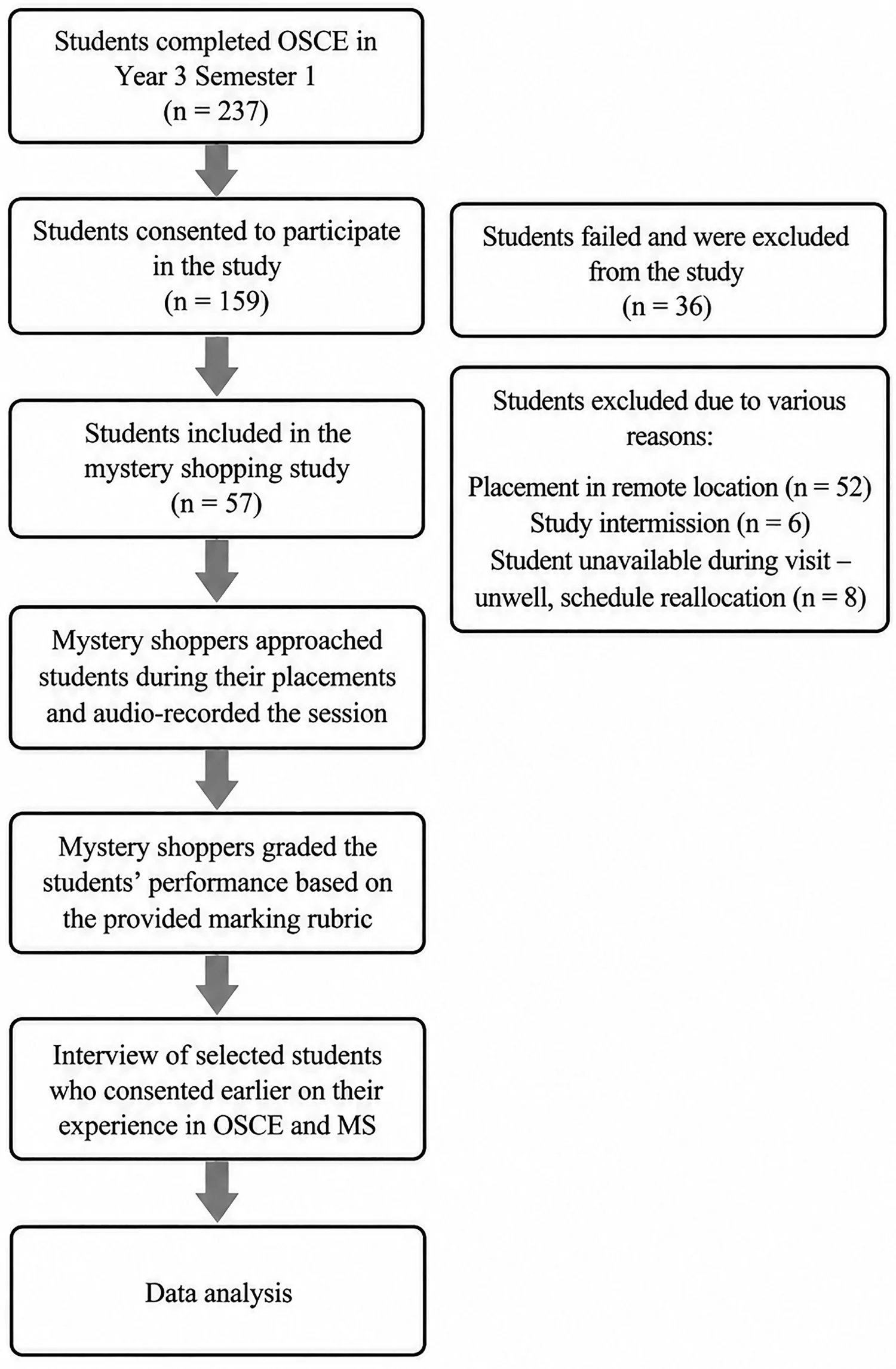
Study flow.

From this group, a final sample of 57 students was evaluated using a single, unannounced covert mystery shopping visit, deliberately scheduled during the mid-to-late phase of their two-week active community pharmacy rotation to ensure adequate on-the-job exposure prior to assessment. Selection for a mystery shopping visit combined convenience sampling with practical placement logistics; students placed at more geographically distant (‘far-flung’) community pharmacies were somewhat underrepresented, as coordinating covert shopper visits across greater travel distances was less logistically feasible within the study timeframe. Detailed participant demographic data, including age distributions and specific placement environments across Malaysia, are compiled in Supplemental Appendix 4. As part of the institutional ethical consent protocol, participants were fully informed that a mystery shopper would evaluate them at an unspecified point during their placement, though the exact timing and physical identity of the shopper were tightly withheld to safeguard the covert integrity of the encounter. Students who failed the university OSCE on their initial attempt were excluded from the analysis pool; because these individuals received targeted remedial support, their inclusion would have introduced a confounding bias and failed to accurately mirror skill transfer from the standard curriculum.

### 2.3. Data collection: mystery shopping procedure

Real-world clinical execution was evaluated in situ through a covert mystery shopping framework (Lim et al., 2023; So et al., 2026; Watson et al., 2009). Although the university examination originally utilised three distinct stations, this study focused exclusively on replicating the primary care station. This specific environment provided the only authentic clinical setting directly and safely accessible to external mystery shoppers without disrupting hospital protocols.

The mystery shoppers were external individuals extensively trained using the identical clinical scripts and case parameters developed for the university OSCE. To minimise the Hawthorne effect and observer bias (Paradis & Sutkin, 2017), the shoppers remained completely unknown to the student cohort, and the operational timeline of the visits was kept undisclosed. Over the two-week rotation block, mystery shoppers collaborated discreetly with site preceptors to verify student shift schedules without alerting the participants. Shoppers then entered the pharmacy incognito, presenting themselves as standard retail customers. As part of the mystery shopping protocol specifically, and distinct from preceptors’ usual active supervisory practice during placement, preceptors were explicitly instructed to step back and allow students to handle this particular customer interaction entirely on their own. Any proactive preceptor intervention to correct, complete, or take over the consultation automatically triggered an immediate ‘failure’ for that specific clinical competency, as it signalled a lack of independent practice readiness.

These covert encounters were audio-recorded, and student performance was mapped against the original OSCE analytical checklist. The communication rubric was omitted from the mystery shopping analysis, as the audio-only recordings did not reliably capture the non-verbal cues (e.g. facial expressions, posture) needed for a fair communication assessment; scoring was therefore restricted to objectively verifiable clinical tasks. Both instruments applied a binary pass/fail threshold to safety-critical checklist items, consistent with common practice in clinical competency assessment where such items cannot be compensated for by strong performance elsewhere; this scoring architecture was applied identically across the OSCE and mystery shopping assessments to preserve direct comparability between simulated and real-world performance.

Following their mystery shopping encounters, students participated in individual, semi-structured post-placement interviews. These debriefing sessions allowed students to reflect openly on the clinical nuances of managing a live case compared to the sterile, predictable environment of the university examination. This qualitative arm was explicitly structured to evaluate the students’ personal encounter with cognitive load and situational anxiety within an authentic practice setting.

To round out the assessment profile, longitudinal EPA scores were extracted directly from the students’ two-week practice logbooks. These evaluations, logged by site preceptors, offered an additional measure of a student's day-to-day readiness to complete core professional duties (e.g. clinical triage, history-taking, and product recommendation) within routine workflow conditions. This supervisor-logged EPA data allowed the study to determine how closely traditional, visible workplace assessments align with completely covert, independent mystery shopping outcomes.

By synthesising data across these distinct dimensions, simulation-based examinations, covert workplace-based mystery shopping, and longitudinal preceptor-led EPA logs, the study deployed a triangulation approach. This multi-layered framework provides a broader assessment of clinical competence, examining how well skills demonstrated in a structured environment transfer to independent practice.

### 2.4. Data analysis

#### 2.4.1. Quantitative data analysis

Quantitative data were processed using Microsoft Excel 2019 (Microsoft Corp, Redmond, WA, USA). The baseline normality of the data distribution was evaluated using the Shapiro–Wilk test. Because the resulting data violated normal distribution assumptions, non-parametric statistical methods were utilised across the board. Specifically, the Wilcoxon Signed-Rank Test was selected to measure the significance and direction of the differences between the paired assessment scores. To evaluate the predictive strength of the relationship between simulated environments and real-world performance, Spearman’s rank correlation coefficient (*r_s_*) was calculated. For all statistical analyses, a threshold of *p*-value < 0.05 was established for statistical significance.

#### 2.4.2. Qualitative data analysis

Consistent with a sequential explanatory mixed-methods design, quantitative results were analysed first and directly informed the qualitative sampling strategy. The impact of the environment on student performance was assessed qualitatively, through students’ self-reported experiences during these interviews, rather than through direct, independent observation of workplace conditions (e.g. pharmacy footfall, interruption frequency) at the time of each mystery shopping visit. For the qualitative arm, students were interviewed sequentially via purposive sampling, selected to include a range of performance tiers across both the university OSCE and the covert field encounters, until no new conceptual insights emerged across successive interviews; this was reached after five interviews. We note that saturation here reflects the specific themes explored within this small purposive sample, rather than saturation of the full range of possible student experiences across the wider cohort (see Limitations). Interviews were conducted via Zoom by authors AB and HYL using a pilot-tested, semi-structured discussion guide (Supplemental Appendix 6). Each debriefing session lasted between 30 and 45 min, was audio-recorded with explicit student consent, and was subsequently transcribed verbatim manually by a member of the research team, rather than through automated transcription services, to keep audio data processing entirely in-house and to maximise transcription accuracy. All transcripts were thoroughly cross-checked for accuracy by a second member of the research team. Adhering to the established six-phase thematic analysis framework outlined by Braun and Clarke (2006), researchers AB and HYL independently coded the text. These codebooks were iteratively compared, and any interpretative discrepancies were resolved through consensus discussions; where agreement could not be reached between the two coders, a third researcher independently reviewed the disputed coding against the original transcript to arbitrate a final decision. This arbitration role was undertaken by SK during the initial coding phase, and subsequently by LLY following a change in author team composition, to establish the final, overarching theme structure.

### 2.5. Ethical considerations

This study received full institutional clearance from the Monash University Human Research Ethics Committee (Project Code: 37796) and was conducted in strict adherence to the ethical tenets of the Declaration of Helsinki. Written informed consent was secured from every participant prior to launching data collection. To prevent any perception of academic pressure or coercion, students were explicitly reminded that participation was entirely voluntary and that opting out or withdrawing at any point would have absolutely zero bearing on their academic grades, progression, or standing within the faculty. To prevent any possibility of research findings influencing academic assessment, mystery shopping performance was never disclosed to course examiners, preceptors, or any individual involved in assigning students’ official academic grades. Prior to anonymisation, participant data, including linked OSCE and EPA records, were separated from the university's academic records system and stored in a folder accessible only to the research team.

To enable comparison of each student's OSCE, mystery shopping, and EPA marks while preserving confidentiality, each participant was assigned a unique study code at the point of data collection; this code, rather than any personally identifying information, was used throughout data analysis and reporting. All collected data were thoroughly anonymised. Any personal identifying details linking data to specific students, preceptors, or pharmacy premises were permanently stripped during the transcription and coding phases to protect identity confidentiality. Finally, raw audio files from both the covert shopper encounters and the student interviews were downloaded and removed from Zoom's cloud storage, then encrypted and safely housed on a secure, password-protected university server accessible exclusively to the primary research team.

## 3. Results

### 3.1. Quantitative comparison: simulated vs. real-world performance

A total of 57 students were evaluated via a single covert mystery shopping visit during their active community pharmacy rotations. The performance disparity across these two settings was statistically substantial: while the cohort achieved a high median analytical OSCE score of 91.67% [IQR:85%−96%], their median mystery shopping score dropped to 0% [IQR:0%−16.7%]. This sharp performance decline was significant across the entire cohort (Wilcoxon signed-rank test, *p* < 0.001). Figure 2 illustrates the divergence in score distributions between the simulated examination and the authentic practice setting. Although the median mystery shopping score was 0%, scores ranged from 0% to 57.14%, with 22 of the 57 students (38.6%) achieving a non-zero score, indicating that a meaningful subset of students were able to independently complete at least some clinical and safety-critical steps without preceptor intervention. The full dataset containing individual student marks across both the 2022 and 2023 cohorts is compiled in Supplemental Appendix 5.

**Figure 2.**
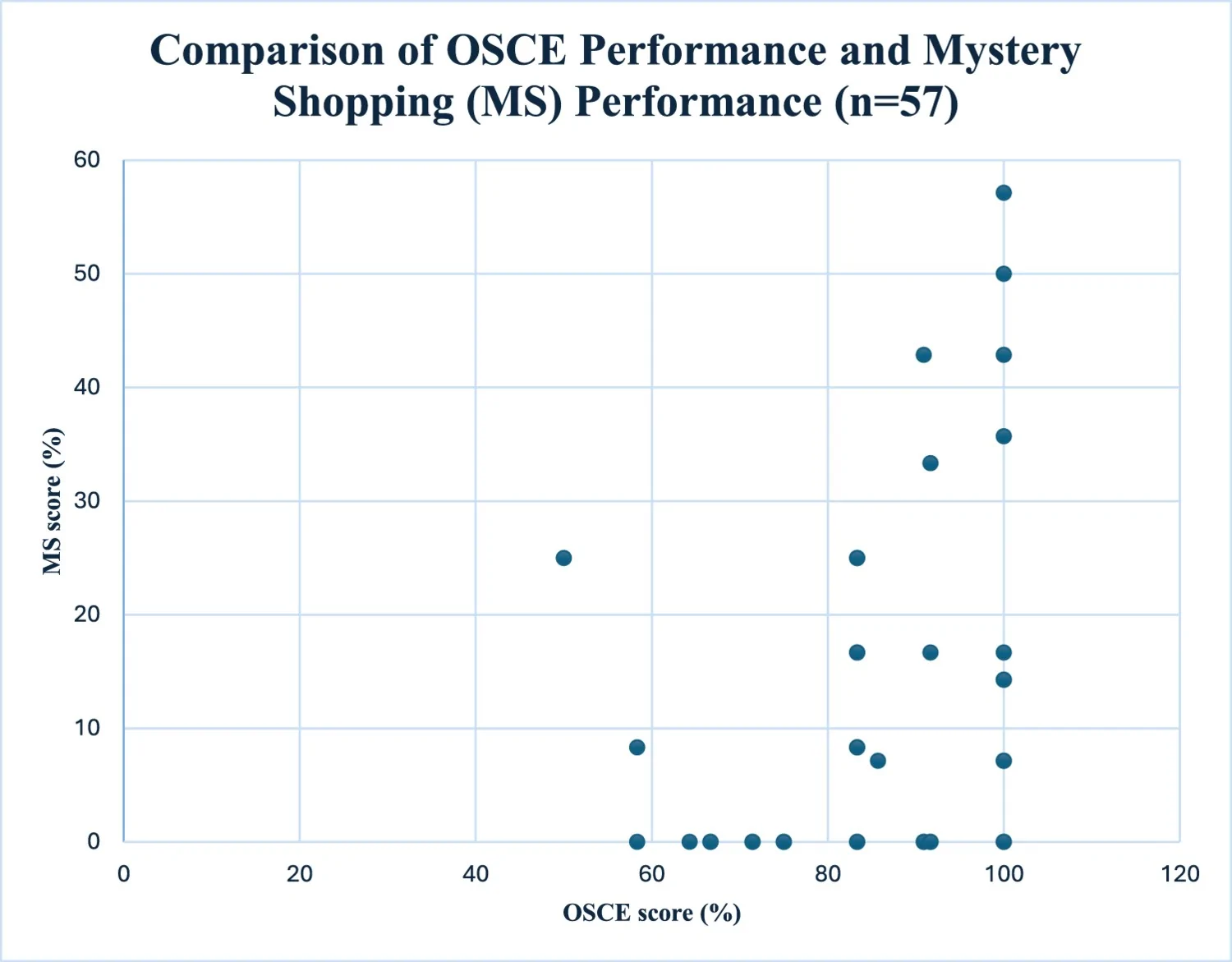
Comparison of third-year pharmacy students’ OSCE and mystery shopping (MS) scores (*n* = 57).

To evaluate whether high achievement within a protective, simulated environment reliably predicted success under real-world conditions, students were stratified into three performance tiers based on their initial OSCE results. As shown in Table 1, the median mystery shopping score remained locked at 0% across Category 1 (90–100%), Category 2 (70–89%), and Category 3 (50-69%), with every comparison achieving statistical significance. Furthermore, a Spearman’s rank correlation analysis confirmed that an individual’s academic success in the OSCE bore no significant relationship to their clinical execution in practice (*r_s_* = 0.14, *p* = 0.2934). This near-absence of correlation should be interpreted alongside the pronounced floor effect in mystery shopping scores (61% of students scoring exactly 0%), which inherently restricts the range of variance available to detect a monotonic relationship, regardless of its true underlying strength.

**Table 1.** Triangulated comparison of student performance (*n* = 57).

Categories^a^	n	OSCE median score (%)	MS median score (%)	*p*-value^b^	Median EPA:(Triage)^c^	Median EPA: (Intervention)^c^
Category 1 (90–100%)	35	100	0	<0.001	Level 4 (Reactive)	Level 4 (Reactive)
Category 2 (70–89%)	16	83.3	0	<0.001	Level 3 (Occasional, Direct)	Level 3 (Occasional, Direct)
Category 3 (50–69%)	6	61.3	0	0.036	Level 3 (Occasional, Direct)	Level 2 (Direct, Proactive)

^a^ Students are categorised based on their initial OSCE results.

^b^ Statistical comparison using Wilcoxon signed-rank tests specifically for OSCE vs. MS median scores; *p* < 0.05 was considered statistically significant.

^c^ EPA Levels: **Level 1**: Observe only; **Level 2**: Perform with direct, proactive supervision; **Level 3**: Perform with occasional direct supervision; **Level 4**: Perform with reactive supervision (quickly available on request)

Abbreviations: EPA, Entrustable Professional Activity; MS, mystery shopping; OSCE, Objective Structured Clinical Examination.

The observed 0% median score in the mystery shopping encounters was driven by a strict binary grading architecture applied to non-negotiable primary care safety standards. In these unscripted field interactions, any omission of core clinical thresholds, such as failing to independently rule out high-risk ‘red flag’ symptoms or neglecting to actively verify patient allergies before arriving at an antimicrobial recommendation, was marked as a fundamental clinical omission. Rather than indicating a total failure to communicate, this floor effect suggests that when supervisor support is removed, students may prioritise rapid over-the-counter product transactions over completing the sequential safety checks required for independent clinical practice.

### 3.2. Qualitative insights into the performance gap

Thematic analysis of the follow-up interviews provided essential context for this quantitative decline, revealing that the structured, rehearsed format of the OSCE failed to equip students for the fluid and unpredictable mechanics of daily practice.

#### 3.2.1. Theme 1: real-life practice isn’t what we imagined

Students reflected deeply on how the rigid, milestone-driven layout of university examinations contrasted with actual clinical practice. One student (Student 1, age 23) noted:

For OSCE, my friends and I will perform role-play to prepare for the OSCE and will go through the checklist. But in real life, we do not have much time for that. But sometimes, we will have short discussions after counselling to discuss the recommendations given.

Another participant admitted that the artificial nature of examinations shifted their learning priorities:

‘OSCE is very structured, and there are certain criteria to meet.’ (Student 3, age 23)

This examination-centric focus tends to build expectations of professional autonomy that do not align with workforce realities. While university curricula train students to act as independent primary care triage leads, the community retail landscape actively restricts this clinical independence. Students frequently found they could not fully utilise their clinical skills, discovering that local community pharmacists do not hold the level of decision-making autonomy they had anticipated. This structural constraint was highlighted by one participant, who described the practical limitations of working within a strict, supervisor-dependent hierarchy:

‘ … When I am giving advice to real-life patients, I feel a bit scared that I might give some wrong information. So, I tend to confirm with my preceptor … ’ (Student 2, age 21)

This shift was intensified by the intense multitasking required in the retail setting:

‘I think definitely there is a difference, because you need to handle many things at the same time in pharmacy; whereas in OSCE we have only the examiner to focus on.’ (Student 3, age 23)

These reflections reveal a clear disconnect between the examination room and the pharmacy setting. The methodical, step-by-step approach expected in an OSCE simply does not fit the fast-paced, retail reality of Malaysian community pharmacies, where daily workload leaves little room for thorough clinical consultations.

#### 3.2.2. Theme 2: real-life distractions affect performance

Unlike the sterile, distraction-free environment of the OSCE, students reported that the operational workload of a busy community pharmacy actively disrupted their ability to apply clinical skills. One participant (Student 2, age 21) remarked:

‘Sometimes the pharmacists and us will be occupied by phone calls or attending to customers who were not really asking for medication – like those wanting cosmetic products. So, I think this will be harder for pharmacists to provide comprehensive care.’

Another student highlighted the logistical strain of peak retail hours:

‘There are many customers, especially during lunchtime. So, it is very hard to attend all at once. We need to focus on one customer at a time, and I think this also affects how we interact with our customers.’ (Student 3, age 23)

Compounding these multitasking demands was a distinct lack of physical privacy, which emerged as a major structural barrier to effective patient communication. Without dedicated counselling rooms, students felt exposed during clinical triage:

‘In retail pharmacy, it is an open area. When I have to suggest products to customers, potentially other customers can overhear our conversation. The privacy would be less in real-life community pharmacies here.’ (Student 5, age 21)

These challenges point to a clear structural barrier in Malaysian community pharmacies: the busy retail environment and the lack of mandated private counselling rooms simply don't allow students to perform the thorough, confidential assessments expected of them in examinations. Notably, students had already completed an earlier, shorter placement within a similar retail pharmacy setting prior to this rotation, suggesting that these coping difficulties reflect a persistent structural challenge specific to the retail environment, rather than simply a lack of prior placement exposure.

#### 3.2.3. Theme 3: public perception limiting real-life performance

Students observed that entrenched patient expectations frequently cut their clinical consultations short. As one student candidly shared:

‘In real-life practice, we do not usually ask them many questions because we do not want to annoy the patients.’ (Student 2, age 21)

Another participant echoed this sentiment, contrasting university clinical requirements with the brisk pace of retail commerce:

‘In OSCE, we are expected to give a lot more details in our counselling points. But in the community pharmacy, it is just shorter – I suppose that is the nature and the pace of the pharmacy.’ (Student 4, age 22)

A third student noted that public impatience actively suppressed thorough history-taking:

‘If you have to ask about all the medical history and medical conditions in real life, it will take a longer time, and most of the customers will not have the patience.’ (Student 5, age 21)

Yet, despite these pervasive environmental pressures, students emphasised that the supportive presence of their preceptors was vital to their learning. They highly valued the chance to interact with live patients within a lower-stakes environment than a university examination:

‘The preceptor and pharmacy assistants here are quite friendly. They are also willing to help out whenever I find difficulties – such as finding the location of medicines or recommendations to patients.’ (Student 1, age 23)

This protective guidance allowed for a gradual, supported transition toward professional autonomy:

‘They let us attend to the patients ourselves, but we need to consult the preceptor before giving any recommendation. This gives us the opportunity to try and learn from our mistakes.’ (Student 3, age 23)

Another student clarified how this balance of independence and oversight functioned in practice:

‘For the most part, I do the history-taking independently. Only when it comes to the final recommendation, I need to get the supervisor’s approval to dispense it. Mostly independent, unless something I am not sure, then I will refer to my supervisor.’ (Student 4, age 22)

These reflections point to two overlapping systemic barriers. Because the broader healthcare system lacks dispensing separation, patients are conditioned to expect fast, retail-style service, a pressure that forces students to sacrifice clinical depth for speed. At the same time, their natural reliance on preceptors creates an artificial safety net. While this supervision is supportive, it may mask whether a student is ready to practice independently without supervision.

### 3.3. Entrustable Professional Activities (EPA) data

This marked discrepancy between independent covert execution and closely supported workplace performance is further validated by the cohort's longitudinal Entrustable Professional Activities (EPA) data. As mapped out in Table 1, most students successfully secured moderate-to-high levels of trust from their preceptors during routine placement blocks. Specifically, 49% of the cohort achieved Level 3 entrustment for primary care triage, while 51% reached Level 3 for identifying clinical interventions, confirming that supervisors trusted them to execute these essential operations with only reactive oversight. For the task of processing prescriptions, 42% reached Level 3, whereas a small 5% remained restricted to Level 1 (observation only).

These metrics indicate that students were able to manage their day-to-day duties within familiar, supervised parameters. However, their performance declined when confronted with the unscripted, spontaneous demands of the covert mystery shopping encounters. While traditional EPA logs capture student clinical performance with preceptor presence, potential intervention, and supervisor feedback, covert mystery shopping examines how students respond independently when this support is removed. This performance mismatch highlights why workforce registration policy must strike a better balance between structured, supervisor-dependent evaluations and unstructured, independent assessments to obtain a genuine picture of clinical readiness.

## 4. Discussion

These quantitative and qualitative findings suggest that the observed lack of independent clinical readiness is unlikely to be explained solely by a deficit in students’ underlying clinical knowledge. Students are extensively taught the relevant content, and their high OSCE scores demonstrate that this knowledge can be reliably demonstrated under structured, supervised conditions. Rather, the qualitative themes presented in Section 3.2 indicate that this gap is primarily driven by environmental and contextual factors, including time pressure, lack of privacy, public expectations, and the removal of preceptor support, compounded by learner-related factors specific to students’ current stage of training, namely their limited prior experience applying this knowledge independently and without oversight. Sections 4.1 through 4.5 unpack these environmental, entrustment-related, and systemic contributors in turn, triangulating the quantitative performance gap with the qualitative themes identified in Section 3.2 to build a converging explanation of this transfer gap, consistent with the triangulation approach outlined in our Methods.

### 4.1. The simulation-to-practice gap: ‘showing’ versus ‘doing’

The primary finding reveals a substantial disconnect between simulated competence and real-world execution. Despite achieving a high median analytical OSCE score of 91.67%, the same student cohort recorded a median mystery shopping score of 0%. This marked divergence provides empirical support for Miller’s Pyramid of Clinical Competence, illustrating that a candidate's verified ability to ‘show how’ within a structured examination does not automatically guarantee the capacity to ‘do’ during an unscripted patient encounter (Miller, 1990). While OSCEs remain valuable for measuring standardised technical skills, these results suggest that performance under simulated conditions may not fully reflect competence under the cognitive load, distractions, and spontaneity of an authentic practice setting (Epstein & Hundert, 2002; Hodges, 2003).

This divergence is also partly attributable to structural differences in how each instrument aggregates checklist performance into a final score. The OSCE composite score reflects a student's cumulative performance across items spanning three separate assessment stations, meaning a single missed safety-critical item is diluted across a broad base of correctly performed items; for illustration, a score of 91.67% is mathematically equivalent to missing just one item out of a twelve-item checklist. In contrast, the mystery shopping score reflects performance within a single, standalone clinical encounter; a missed safety-critical item within this smaller item pool therefore has a proportionally much larger impact on the overall score and can reduce the encounter score to zero where that item is treated as a critical failure. High median OSCE scores should therefore be interpreted with this scoring architecture in mind, rather than solely as evidence of uniformly high underlying competence.

This ‘extrapolation gap’ becomes even more pronounced when contrasting completely independent performance against standard, supervised workplace benchmarks. Interestingly, the longitudinal Entrustable Professional Activity (EPA) records show that nearly half of the students achieved Level 3 entrustment, a tier traditionally interpreted as achieving the autonomous ‘Does’ level under routine conditions. However, the discrepancy between high supervisor ratings and the mystery shopping floor effect suggests that the presence of a preceptor may provide an important safety net. Once this support was removed during the covert mystery shopping interactions, important clinical omissions became apparent, suggesting that competence demonstrated under supervision may not fully reflect independent clinical performance.

Students who achieved non-zero scores typically completed a substantial portion of the history-taking and counselling process independently, navigating the encounter without requiring reactive preceptor intervention for at least some competencies. This suggests that partial independent competence is achievable at this stage of training. One plausible driver of this variability is the specific clinical scenario encountered, as the three mystery shopping cases differed in complexity; however, case-level analysis was beyond the scope of the present study, and future research should examine whether scenario-specific factors influence independent performance.

This transfer gap likely reflects not only contextual pressures but also learner-related factors specific to students’ current stage of training. As third-year students, participants were applying previously taught clinical knowledge independently, without preceptor oversight, for the first time; this transition from supervised to unsupervised practice is itself a normal part of professional development, and some hesitancy or inconsistency in performance is an expected feature of this developmental stage rather than a deficit in prior teaching. The following sections consider how these learner-related factors interact with, and are compounded by, the environmental and systemic pressures identified in our qualitative data.

### 4.2. Environmental constraints and the ‘priority shift’

Qualitative data suggest that this observed transfer gap is substantially driven by a fundamental shift in student priorities when transitioning from simulation to actual practice. In the distraction-free university examination, students are entirely assessment-centric, focusing their cognitive energy on satisfying specific checklist parameters. In the live community pharmacy setting, however, their focus is split by acute patient demands and immediate situational constraints. Students reported that the busy, multitasking environment of a community pharmacy, such as managing phone lines or triage requests for non-medicinal cosmetic products, adds operational demands that an OSCE simply cannot reproduce.

Similar challenges have been reported elsewhere. International evidence highlights limitations in antimicrobial dispensing practices within community pharmacies, including gaps in patient questioning and counselling (de Souza et al., 2022). This operational strain was mirrored by our participants, who noted that a perceived lack of acoustic privacy in the open retail setting and an overriding desire not to annoy customers who expect rapid service compelled them to favour commercial speed over clinical depth.

This tension is particularly visible within the communication domain. Providing structured, high-quality counselling becomes more difficult when navigating the fluctuating patient expectations and language variations inherent to a multicultural retail landscape (McCann et al., 2023). As a result, the communication rubric was omitted from the final mystery shopping analysis, as these fluid paralinguistic and situational nuances could not be reliably or fairly captured via covert audio recordings. This deliberate omission shifts the research focus strictly to objective clinical safety thresholds, suggesting that the fast-paced community pharmacy setting may restrict the methodical communication patterns expected in university simulations. These environmental factors may limit students’ ability to apply their academic training, contributing to omissions in baseline patient triage and safety checks.

### 4.3. The preceptor ‘safety net’ and entrustment discrepancies

The discrepancy between independent mystery shopping performance and preceptor-logged EPA milestones is an important finding of this study. While independent clinical execution declined to a 0% median during covert audits, roughly half of these same students routinely received Level 3 entrustment ratings from their workplace supervisors. This indicates that within standard placement operations, they were explicitly trusted to perform primary triage with only reactive oversight. As Murphy et al. highlight, while workplace-based assessments (WBAs) are essential for capturing clinical behaviour in situ, they are inherently compromised by the visible presence of an evaluator (Murphy et al., 2009a, 2009b).

Our interviews show that this supervisor-dependent design creates a systematic bias. Students routinely defaulted to their preceptors for final recommendations, out of a legitimate fear of giving incorrect clinical advice to a live patient. This constant reliance on supervisor sign-off aligns with Clouder et al.’s work on professional autonomy, which outlines how students carefully balance a desire for independence against the psychological safety of a workplace hierarchy (Clouder et al., 2022). Consequently, preceptors often rate a student as competent because they are evaluating the corrected ‘final result’ of a joint consultation. Conversely, the covert mystery shopper observes the student independently, capturing clinical omissions that may occur when supervisor oversight is removed.

This distinction between independently demonstrated and preceptor-supported performance carries implications for experiential assessment design beyond the Malaysian context. Many workplace-based assessment frameworks, including EPA-based entrustment models, implicitly assume that supervised performance ratings reflect a trajectory toward independent competence; our findings suggest these two constructs may diverge substantially once supervision is fully removed. Programmes relying solely on supervised evaluations to certify practice-readiness may therefore be at risk of overestimating students’ unsupervised capability, a consideration relevant to workplace-based assessment reform more broadly.

### 4.4. Systemic barriers and the Malaysian context

The Malaysian healthcare infrastructure presents unique, long-standing systemic challenges that actively inhibit the transfer of clinical skills from the university to the pharmacy setting. The primary driver of this friction is the persistent practice of physician dispensing within the private sector. As outlined by Shafie et al. (2012) and reaffirmed by Tiong et al. (2016), the historical absence of dispensing separation (implemented only within government hospitals under Malaysia's two-tier healthcare system) effectively relegates private-sector community pharmacies to a retail-dominated role, substantially limiting opportunities for pharmacists to perform complex clinical reviews. This structural limitation is worsened by the public perception realities identified by Abdul Ghani et al. (2021), which show that community awareness and acceptance of dispensing separation remain exceptionally low. Patients are culturally conditioned to favour one-stop medical clinics and frequently view the additional step of consulting a community pharmacist as an unnecessary inconvenience.

This systemic reality shaped our qualitative findings. Students noted that consumers consistently treat the pharmacy as a destination for rapid, transactional product purchasing rather than an accessible primary care site. Furthermore, research by Chu et al. (2024) and Loh et al. (2021) confirms that Malaysian community pharmacists face significant operational constraints, including high commercial workloads and public biases that favour physicians for all clinical advice. These external pressures directly shrink the student’s operational boundaries on placement; if the local practice ecosystem does not actively demand or respect pharmacist-led clinical interventions, students are rarely given the space to exercise the independent decision-making tested in our mystery shopping encounters.

Despite this systemic friction, we maintain that practice-readiness remains a valuable and necessary educational goal. Pharmacists continue to perform essential clinical safety functions, such as allergy screening, drug interaction checks, and appropriate self-care triage, regardless of who initiates the original prescription, and these functions are directly reflected in the competencies assessed in this study. Demonstrating robust independent clinical competence is also a prerequisite for any future expansion of pharmacists’ scope of practice within the Malaysian healthcare system. We acknowledge, however, that the prevalence of physician dispensing remains an ongoing and unresolved structural challenge for the profession in Malaysia, and that strengthening practice-readiness should be pursued alongside, rather than as a substitute for, continued advocacy for dispensing separation reform.

Finally, our specific focus on antimicrobial dispensing adds a final layer of regulatory friction. Community-level dispensing of antimicrobials is notoriously error-prone worldwide (de Souza et al., 2022). Within the Malaysian context, although third-year undergraduates are extensively taught prescription validity screening as part of the curriculum, their community pharmacy placement represents the first opportunity to apply this knowledge independently, without direct preceptor oversight. This gap between taught knowledge and independent execution may contribute to students adopting a more cautious, passive role (Abdul Ghani et al., 2021). While this hesitation is a safe and responsible reaction to the unfamiliarity of unsupervised practice, it may also have contributed to the low independent performance scores captured by our covert shoppers. Empowering students will require clearer professional scopes, as well as structural and policy reforms, to ensure academic training aligns with genuine, real-world practice opportunities.

### 4.5. Regulatory and policy imperatives: embedding independent audits into national quality assurance

While educators have long agreed that a single examination cannot judge professional competence, these findings push the conversation from simple classroom pedagogy into the realm of national workforce regulation. The fact that independent performance declined to a 0% median score raises important questions about the extent to which current licensing and accreditation processes capture students’ true independent capability. Relying too heavily on predictable, supervised milestones, such as OSCEs or preceptor-signed logbooks, can build an artificial safety net around candidates that masks what they are capable of doing on their own (So et al., 2026).

To address this important gap, regulatory bodies like the Pharmacy Board of Malaysia may wish to consider whether independent, covert quality audits could complement existing credentialing pathways, pending further validation, particularly during the Provisional Registered Pharmacist (PRP) training year. Systemic deployment of a mystery shopping framework offers a more direct view of autonomous practice, uncovering critical safety omissions that standard supervisor-led check-ins miss (Boursicot et al., 2011). Building a practice-ready workforce may require looking beyond curricula alone to also consider the commercial pressures of the retail environment, though this remains a direction for future policy discussion rather than a conclusion this study can establish. Coordinated engagement among pharmacy owners, professional bodies, and universities may be one avenue worth exploring to better support high-quality clinical care within the retail pharmacy setting (FIP, 2025). Complementing regulatory and curricular reforms, targeted public health campaigns to re-educate patients on the clinical role of community pharmacists, distinct from their perception as retail product providers, may also help shift patient expectations toward supporting the kind of thorough clinical consultations this study's findings suggest are currently discouraged by prevailing retail-oriented patient behaviour (see Theme 3). Beyond the immediate Malaysian context, these findings may offer preliminary insights relevant to other developing health infrastructures, particularly where community pharmacy practice remains closely tied to retail dispensing; however, given the single-institution, single-country setting and the relatively early stage of training of our participants, further research across diverse settings and training levels would be needed before these implications could be generalised.

## 5. Strengths and limitations

This study offers a distinct contribution to the global pharmacy education literature by empirically testing the utility of mystery shopping to evaluate real-world skill transfer, particularly in the Malaysian and broader Southeast Asian contexts. Historically, these regions have relied almost exclusively on standard, simulation-based examination matrices; our data regarding the operational feasibility and value of mystery shopping may inform future discussions on regional workforce policy and assessment reform by demonstrating how to bridge simulation with real-world quality assurance. A core strength of this research lies in its sequential mixed-methods architecture, using semi-structured qualitative interviews to provide a clear, contextualised look into the marked ‘floor effect’ captured in our quantitative scores.

Several limitations must be acknowledged. First, due to intense logistical constraints and shifting student placement schedules, not every consented student received a covert shopper visit. This resulted in an uneven distribution across our performance tiers, with high-achieving students disproportionately represented in the 90%–100% baseline OSCE bracket. While this distribution accurately reflects the high-performing nature of this specific cohort, it may limit the immediate generalisability of these findings to students with lower baseline simulation scores. This selection process also combined convenience sampling with practical placement logistics, resulting in some underrepresentation of students placed at more geographically distant pharmacies, which may further limit the generalisability of these findings to students in remote or far-flung placement settings. Second, institutional variables, such as strict corporate site policies or particularly protective preceptor oversight, may have restricted student autonomy in practice, potentially under-representing their true standalone capabilities. Third, the clinical scope was explicitly bound to infectious disease triage; therefore, these trends may vary across other chronic disease states.

Fourth, the binary scoring architecture, while methodologically consistent across both assessment modalities, does not distinguish between a near-complete consultation with a single critical omission and one demonstrating minimal competence throughout; this may understate the granularity of students’ actual performance, and future work could explore a graduated scoring approach for non-safety-critical items.

Fifth, our qualitative findings are based on a small purposive sample of five students and should be interpreted as illustrative rather than representative of the broader cohort's experiences; while thematic saturation was observed within this sample, a larger or more diverse qualitative sample may have surfaced additional perspectives not captured here.

Sixth, the communication rubric was omitted from the mystery shopping analysis, as the audio-only recordings did not reliably capture the non-verbal cues needed for fair communication assessment; as a result, our findings do not capture the full scope of communication competencies students may have demonstrated during these encounters.

Seventh, our assessment of environmental influence relies on students’ self-reported experiences rather than independent, objective observation of workplace conditions at the time of each mystery shopping visit; future studies could strengthen this by incorporating structured environmental observation alongside covert assessment.

Future research should deploy this covert methodology across a broader range of clinical conditions to test if these observations hold across different therapeutic areas. Where universal implementation is logistically constrained, a targeted mystery shopping approach, potentially incorporating telehealth or digital e-commerce, could offer a scalable, flexible audit tool to track performance gaps and guide national curriculum adjustments.

## 6. Conclusion

The integration of a covert mystery shopping framework in this study exposes a marked disconnect between pharmacy students’ simulated examination performance and their capacity to apply those same clinical skills in an unscripted practice environment. Even when confronted with primary care scenarios completely identical to their university simulations, the candidates’ clinical thoroughness declined, yielding a median independent performance score of 0%. These outcomes, together with the qualitative findings, illustrate how local healthcare structures, public expectations, and retail workplace realities may constrain a student's independent clinical execution.

Bridging this ‘transfer gap’ may require shifting away from isolated educational interventions and moving toward broader, system-wide policy reforms. Developing a practice-ready workforce will require coordinated efforts among academic institutions, pharmacy professional bodies, and community pharmacy stakeholders to legally and operationally realign undergraduate clinical training with the fast-paced realities of modern community practice. Student 2's reported hesitancy to act without preceptor confirmation further illustrates that the OSCE format, as currently structured, may not capture whether a student can exercise sound clinical judgement in the absence of an authority figure to defer to, a capability distinct from, but conflated with, checklist-verified clinical knowledge. At an institutional level, these findings also suggest a more immediate, actionable step: incorporating greater unpredictability and time pressure into OSCE design and checklist preparation, rather than relying solely on structured, rehearsable formats, may better prepare students for the fluid nature of real-world practice. Ultimately, these preliminary findings suggest that independent, covert workplace audits may merit further investigation as one component of national registration and quality assurance frameworks, though larger, multi-site studies involving students at later stages of training would be needed to establish their value before any policy adoption.

Future research should build on these findings by examining the influence of case-specific factors on independent performance, exploring graduated scoring approaches for non-safety-critical competencies, and incorporating structured environmental observation alongside covert assessment, to further refine our understanding of the transfer gap identified here.

## Declarations

## Supplementary Material

Appendices Mystery Shopping FINAL.docx

## Data Availability

Due to institutional ethics restrictions protecting participant confidentiality, the primary datasets are not publicly available. De-identified summary metrics may be requested from the corresponding author.
