# Supplementary material for: Rethinking assessment for practice readiness: policy implications of the OSCE–practice mismatch in Malaysia
Source: J Pharm Policy Pract. 2026 Sep 30;19(1):2735132. doi: 10.1080/20523211.2026.2735132 (PMC13629800; doi:10.1080/20523211.2026.2735132)
Supplement: Supplementary file 1 [file JPPP_A_2735132_SM3283.docx]

**Appendices**

**Appendix 1: General Analytical Checklist**

This analytical checklist was consistently applied to evaluate performance in both the university-based Objective Structured Clinical Examination (OSCE) and the audio-recorded workplace-based Mystery Shopping (MS) encounters, ensuring direct comparability across settings.

| **Gathering Information/Introduction** | **Yes** | **No** |
| --- | --- | --- |
| 1. Elicits who the medication for |  |  |
| 2. Elicits the indication of the medication(s) |  |  |
| 3. Elicits the past medical history of the patient |  |  |
| 4. Elicits the past medication history of the patient |  |  |
| 5. Elicits the over-the-counter medication(s) or herbal supplement(s) |  |  |
| 6. Elicits the drug allergy and nature of allergy of the patient |  |  |
| 7. Elicits the pregnancy and breastfeeding status of the patient |  |  |
| **Management Strategies/Patient Education** | **Yes** | **No** |
| 1. Counsels the patient correct administration dosage, frequency and duration |  |  |
| 2. Counsels the patient to take the medication on an empty stomach or with food |  |  |
| 3. Counsels the patient the precautionary measures while taking the medication |  |  |
| 4. Counsels the patient on the common side effects of the medication |  |  |
| 5. Recommends the patient non-pharmacological advice for the disease |  |  |
| **Monitoring/Follow Up** | **Yes** | **No** |
| 1. Advises the patient to see their doctor /pharmacist if their symptoms do not improve or worsen |  |  |

**Appendix 2: Summary of Assessment Scenarios**

The mystery shopping encounters utilized standardized clinical scenarios identical to those encountered by students during their university-based OSCEs. All scenarios were grounded in infectious disease management, requiring students to demonstrate core competencies in history-taking, clinical reasoning, and patient education.

**Case 1: Complex Prescription Validation (Amoxicillin in Pregnancy)**

The scenario focused on complex prescription validation involving a pregnant patient prescribed high-dose amoxicillin for community-acquired pneumonia. The student was required to identify a history of penicillin hypersensitivity, specifically a past urticarial reaction to flucloxacillin, and recognise that amoxicillin was contraindicated due to the high risk of cross-reactivity. To meet the critical criteria, students had to inform the customer that the medication was unsafe and refer them back to the prescriber for a safer alternative.

**Case 2: Symptomatic Triage and Referral (Cellulitis/Skin Infection)**

The second scenario involved symptomatic triage and referral for an elderly patient with a suspected skin infection. A customer presented with a request for over-the-counter hydrocortisone cream to treat a "cat scratch," but the clinical history revealed spreading redness, warmth, swelling, purulent discharge, and systemic fever (38.5 °C). Students were expected to identify these diagnostic symptoms of cellulitis and recognize that topical steroids were inappropriate and potentially harmful. The critical criteria for this case required an immediate referral to a physician or hospital to prevent further complications from a systemic infection.

**Case 3: Medication Safety and Drug-Drug Interactions**

The third scenario addressed medication safety and drug-drug interactions (DDI) concerning a ciprofloxacin prescription for a urinary tract infection in an elderly patient. Through a comprehensive medication review, students were expected to elicit that the patient was concurrently taking iron supplements. This required the student to identify the significant interaction between quinolones and multivalent cations, which reduces antibiotic absorption. To pass the critical criteria, the student had to counsel the patient on the necessity of separating the administration of the iron supplement and the antibiotic by at least two hours to ensure therapeutic efficacy.

**Appendix 3: EPA Entrustment Level Definitions**

**Part A: EPA Level Descriptions**

This appendix details the Entrustable Professional Activities (EPAs) used to evaluate student performance during their community pharmacy experiential placements. These assessments provide a measure of workplace competency and the level of supervision required for core professional tasks.

| **EPA Level** | **Description** |
| --- | --- |
| Level 1 | Observe only, even with direct supervision. |
| Level 2 | Perform with regular direct, proactive supervision and intervention. |
| Level 3 | Perform with occasional direct, proactive supervision and intervention. |
| Level 4 | Perform with reactive supervision (i.e., on request and quickly available). |

#### **Part B: Evaluated Pharmacy Activities**

Preceptors assessed students based on their ability to perform the following five core activities. Activities marked with an asterisk (*) are directly relevant to the clinical tasks simulated in the mystery shopping scenarios.

| **Activity** | **Description of Competency** |
| --- | --- |
| ***Primary Care Triage and Provision** | Utilise appropriate processes to obtain patient history, assessment of common conditions seen in community pharmacy, and provide appropriate recommendations/referrals in uncomplicated patients. |
| ***Receiving Prescription/Request** | Demonstrate ability to ask the appropriate clinical/administrative questions when receiving a prescription/request from a consumer. |
| **Complementary and Alternative Medicines** | Assess appropriateness and provide support to consumers using complementary and alternative medicines by using evidence-based decision making. |
| **Use of Device** | Demonstrate ability to use and provide counselling of device, including follow up/monitoring (e.g., inhaler, blood pressure monitor, glucose check). |
| ***Clinical Intervention Identification** | Ability to identify medication-related issues with patients and justify treatment recommendation. |

***Note*:** The EPAs for Primary Care Triage and Clinical Intervention Identification directly correlate with the infectious disease scenarios (penicillin allergy, cellulitis triage, and drug-drug interactions) used in the mystery shopping exercise. While the EPA scores reflect performance in a supported workplace environment, the mystery shopping scenarios evaluate the same competencies in an independent, unscripted context.

**Appendix 4: Participant Demographics**

| **Characteristic** | **Description** |
| --- | --- |
| **Age Range** | 21 – 23 years |
| **Academic Standing** | Year 3 Undergraduate Pharmacy Students |
| **Prior Clinical Training** | Completed 10-week Infectious Disease Module and OSCE |
| **Placement Setting** | Accredited Community Pharmacies in Malaysia |
| **Pharmacy Type** | Mix of independent retail pharmacies and corporate chains |
| **Geographic Context** | Urban regions within West Malaysia |

***Note*:** The demographic profile represents a high-achieving cohort of Year 3 pharmacy students who had successfully completed their theoretical and simulated training in infectious disease management.

**Appendix 5: Students’ Scores from OSCE and MS settings (*n=57*)**

| **Cohort** | **OSCE score (%)** | **MS score (%)** |
| --- | --- | --- |
| **2023**  (*n=34*) | 100 | 0 |
|  | 91.67 | 0 |
|  | 100 | 7.14 |
|  | 100 | 14.29 |
|  | 91.67 | 0 |
|  | 100 | 0 |
|  | 100 | 7.14 |
|  | 91.67 | 0 |
|  | 100 | 0 |
|  | 100 | 0 |
|  | 100 | 0 |
|  | 83.33 | 0 |
|  | 100 | 0 |
|  | 91.67 | 0 |
|  | 91.67 | 0 |
|  | 64.29 | 0 |
|  | 71.43 | 0 |
|  | 71.43 | 0 |
|  | 71.43 | 0 |
|  | 85.71 | 7.14 |
|  | 71.43 | 0 |
|  | 90.91 | 0 |
|  | 100 | 50 |
|  | 100 | 14.29 |
|  | 100 | 0 |
|  | 100 | 0 |
|  | 90.91 | 42.86 |
|  | 100 | 42.86 |
|  | 100 | 14.29 |
|  | 100 | 0 |
|  | 100 | 0 |
|  | 100 | 35.71 |
|  | 100 | 0 |
|  | 100 | 57.14 |
| **2022**  (*n=23*) | 83.33 | 16.67 |
|  | 100 | 16.67 |
|  | 100 | 0 |
|  | 91.67 | 33.33 |
|  | 50 | 25 |
|  | 58.33 | 0 |
|  | 75 | 0 |
|  | 83.33 | 0 |
|  | 58.33 | 8.33 |
|  | 83.33 | 8.33 |
|  | 100 | 0 |
|  | 100 | 0 |
|  | 91.67 | 0 |
|  | 83.33 | 0 |
|  | 83.33 | 25 |
|  | 83.33 | 25 |
|  | 91.67 | 0 |
|  | 83.33 | 16.67 |
|  | 66.67 | 0 |
|  | 66.67 | 0 |
|  | 91.67 | 16.67 |
|  | 75 | 0 |
|  | 83.33 | 8.33 |

***Note:*** For entries where the MS score is >0% (e.g., 50% or 57.14%), students successfully performed a significant portion of history-taking and counselling steps independently. These higher scores indicate instances in which students navigated the encounter without reactive supervisor intervention, which would otherwise have resulted in a score of zero for the relevant competencies.

**Appendix 6: Semi-Structured Interview Guide**

Introduction and information recall

1. We appreciate your participation in the interview. We realize it has been a while since the mystery shopper visits took place. However, we would like to inquire if you recall any specific details from the visits and if you can recall, could you tell when the mystery shopper visited during your placement training? Thank you."

Preparation and self-reflection

2. How did you prepare for your OSCE and placement respectively? What do you think about your readiness and performance when dealing with a case in OSCE and in a real-life situation during your placement?
3. What was your perception of the difference between being evaluated in the OSCE setting and offering advice to a genuine patient in a real-world scenario?

Factors contributing to unequal performance in real-life scenarios and OSCE

4. What do you think about interacting with the preceptor and working colleagues, such as pharmacy assistants in your assigned community pharmacy? Do you find yourself an opportunity to attend to a case and provide recommendations and counselling to a patient during your placement training?
5. Could you describe the pharmacy's environment during placement training regarding the number of customers, noise, and phone calls? Do you think these factors might have influenced your placement performance compared to your OSCE performance?
6. Can you identify any other potential factors that may have impacted your performance in the real-life scenario during placement training as compared to the OSCE?
7. How might you approach a similar situation differently in the future?
